# An analysis of coronavirus disease 2019 with spline regression at province level during first-level response to major public health emergency out of Hubei, China

**DOI:** 10.1017/S095026882000312X

**Published:** 2021-01-05

**Authors:** Chen Liang, Li Shen

**Affiliations:** Shanghai Jiao Tong University Affiliated Sixth People's Hospital, Shanghai, China

**Keywords:** COVID-19, first-level response, major public health emergency, spline regression

## Abstract

This study aims to locate the knots of cumulative coronavirus disease 2019 (COVID-19) case number during the first-level response to public health emergency in the provinces of China except Hubei. The provinces were grouped into three regions, namely eastern, central and western provinces, and the trends between adjacent knots were compared among the three regions. COVID-19 case number, migration scale index, Baidu index, demographic, economic and public health resource data were collected from 22 Chinese provinces from 19 January 2020 to 12 March 2020. Spline regression was applied to the data of all included, eastern, central and western provinces. The research period was divided into three stages by two knots. The first stage (from 19 January to around 25 January) was similar among three regions. However, in the second stage, growth of COVID-19 case number was flatter and lasted longer in western provinces (from 25 January to 18 February) than in eastern and central provinces (from 26 February to around 11 February). In the third stage, the growth of COVID-19 case number slowed down in all the three regions. Included covariates were different among the three regions. Overall, spline regression with covariates showed the different change patterns in eastern, central and western provinces, which provided a better insight into regional characteristics of COVID-19 pandemic.

## Introduction

On 31 December 2019, the WHO China Country Office was informed that cases of pneumonia of unknown aetiology were found in Wuhan City, Hubei Province of China [1–4]. On 7 January 2020, the Chinese authorities identified a new type of coronavirus. And the coronavirus study group of the International Committee on Taxonomy of Viruses named it as severe acute respiratory syndrome coronavirus 2 (SARS-CoV-2) on 11 February 2020 [5]. On the same day, WHO named this new infectious disease as coronavirus disease 2019 (COVID-19) [6].

On 23 January 2020, the Chinese government initiated the first-level response to major public health emergencies. The central and all levels of governments took a combination of measures to prevent and control the outbreak of COVID-19. Until 12 March 2020, the number of daily confirmed cases out of Hubei had reduced to 3. Considering the limited number of active COVID-19 cases, the levels of response to major public health emergency in many regions were adjusted to second- or third level. Until the end of June, only 2750 local cases were newly confirmed out of Hubei, averaging 25 cases per day.

In the meantime, the outbreak of COVID-19 has become a global health threat as more than 200 countries and regions from Africa, America, eastern Mediterranean, Europe, South-East Asia and western Pacific are being influenced by COVID-19. Until 30 June 2020, a total of 10 185 374 cases had been confirmed around the world, including 503 862 deaths [7]. The growing trend of confirmed COVID-19 cases has not been effectively contained globally. Previous studies have reported the aetiological, epidemiological, clinical characteristics and treatment of COVID-19 cases [8–13], but few researchers have analysed the knots of COVID-19 case number with spline regression. After the implementation of controlling measure, the number of COVID-19 cases showed non-linear characteristics and non-trend patterns. Splines allow for smooth transitions and subtle structural shifts by piecing together different polynomial line segments, which is an alternative model to deal with the characteristics of COVID-19 case number. Agiwal's study [14] showed spline function is useful to convert the non-linear trend of newly COVID-19 cases into a linear pattern. Sousa *et al*. [15] proposed an automatic method based on the minimisation of the sum of squared residuals plus a penalty to estimate the knot number and locations. Demertzis *et al*. [16] applied regression splines of random knots and complex-network regression splines to fit cumulative COVID-19 infection, death and ICU patient curve of Greece, which performed better than cubic fittings. In these studies, social and medical factors were not considered, which would influence the spread of SARS-CoV-2. And as far as we know, there were no studies analysing the data of COVID-19 cases in China with spline terms.

Therefore, this study was conducted to analyse the cumulative COVID-19 case number during the first-level response in provinces out of Hubei, who experienced importing, spreading and containing stages during this period. Influencing factors at province level were considered, too. The first confirmed cases in the provinces out of Hubei were all imported cases, indicating the influence of transient population from Hubei. Detection, diagnosis, isolation and treatment of COVID-19 cases heavily relied on the public health resources in each province, and population susceptibility might be influenced by demographic characteristics, economic status and access to COVID-19 information. Stepwise procedure was used to select knot numbers, locations and covariates, which were statistically selected [17]. The objectives of this study were: (1) to locate the knots of COVID-19 case number, (2) to describe the change pattern of each segment; (3) to explore the difference of change patterns among eastern, central and western provinces.

## Methods

### Data collection

The daily numbers of newly confirmed COVID-19 cases between 19 January 2020 and 12 March 2020 for all provinces (except Hubei) were obtained from the websites of Provincial Municipal Health Commission. This 54 day research period was chosen specifically because the first COVID-19 case out of Hubei was confirmed on 19 January 2020 and 26 provinces adjusted emergency response to second- or third level before 12 March 2020. Provinces initiating the first-level response to major public health emergency between 23 January 2020 and 25 January 2020 were included, and provinces with incomplete data or reporting less than 100 cases during the research period were excluded.

Daily transient population size at the province level was obtained from Qianxi Map, an open data platform funded by Baidu [18]. Baidu provides mobile termination service for more than 1.1 billion people and location-based service (LBS) over 120 billion times per day in China. When a customer sends a service request to Baidu, his or her location is included in the request. Therefore, the transient population to any provinces and regions are recognised through the location-based system, no matter what types of transportation they take. Migration scale index, calculated based on LBS data, represents the size of the transient population. It is comparable horizontally among provinces or regions, and one migration scale index is equal to about 56 137 travellers [19]. For each included province, daily migration scale index from Hubei was collected.

Baidu index (BI) of two variances of novel coronavirus and three variances of novel coronavirus pneumonia was used to reflect public attention to COVID-19. This index was calculated based on the number of times that a word appeared in Baidu search engine, which was the largest search engine in China, holding 63.56%–72.72% market share in China from January 2020 to March 2020 [20, 21]. These variances had the highest searching frequency in Baidu search engine among the ones related to COVID-19 during the research period.

Yearly demographic, economic and public health resource data of each province in China were extracted from Chinese Health Statistical Yearbook 2019, published by Chinese National Municipal Health Commission on 1 August 2019 [22]. This book presented Chinese health care and inhabitant's health status in the whole nation and each province in 2018. Provinces were grouped into eastern, central and western, according to the geographic location. Population by age groups (0–14 years old, 15–64 years old and over 65 years old) and sex ratio (male/female) at province level were extracted from this book, since previous studies had reported older age and gender as potential factors affecting the risk of COVID-19 at personal level [23, 24]. Per capita gross domestic product (GDP), per capita health care expenditure, number of registered doctors, nurses, beds in infectious disease department and public health professionals per 1000 population at province level were collected as well, because they probably would reflect economic status related to general health and public health resources related to infectious disease.

### Data analysis

Continuous variables were presented as median and inter-quartile range (IQR). Categorical variables were described as frequencies and percentages. Pearson correlation was conducted to explore the correlation among daily-reported variables, and Spearman rank-order correlation was conducted among annually reported variables. Annually or daily reported variables, among which there was no correlation, would be included in multivariate regression. Interaction terms of included variables would be included, if their effects on containing pandemic might be related. Then, multivariable analysis with spline terms was conducted to fit the number of cumulative COVID-19 cases at province level. In order to explore the possibility of different knots in eastern, central and western regions, four models were built based on data of eastern, central, western and all included provinces, respectively. The general model for *k* knots in an *n* degree polynomial regression was
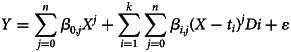
where *Y* was cumulative COVID-19 case number and *X* represented the number of days since 19 January 2020. The *X* values of the *k* knots were designated *t_i_* where *i* = 1,2…*k*. Since the number and location of spline knots were unknown, a series of dummy variables (*D_i_*) were created as follows:
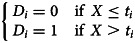
where *t_i_* = 1,2,…,53. Linear (*n* = 1), quadratic (*n* = 2) and cubic (*n* = 3) adjustments were applied, respectively. Stepwise regression was used to select the degree of adjustment, knot locations and covariates. Variance inflation factor (VIF) was used to assess co-linearity. Adjusted R-square (*R*^2^) and Akaike information criterion (AIC) were used to evaluate the performance of the models. All the analysis was performed with Statistical Analysis System (SAS, version 9.4, SAS Institute).

## Results

### Characteristics of provinces out of Hubei

**Fig. 1.**
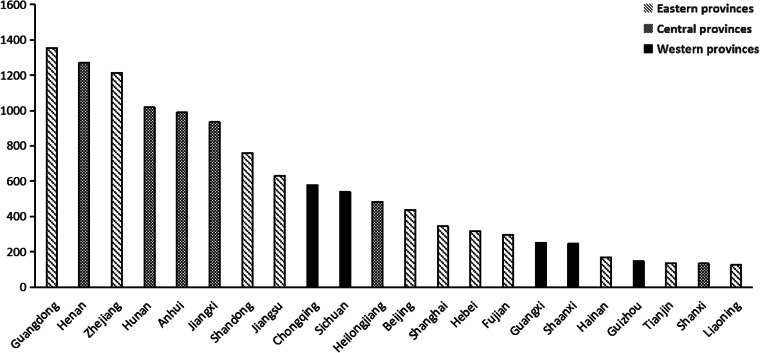
Cumulative number of COVID-19 cases for 22 provinces from 19 January 2020 to 12 March 2020.

Data from 22 provinces in China were analysed in this study. As of 12 March 2020, the median of cumulative COVID-19 case number in the 22 provinces was 459 (IQR: 245–935), while Guangdong, Henan, Zhejiang and Hunan reached over 1000 cases (Fig. 1). Central provinces had a median of 962 cases, when the data for eastern and western provinces were 346 and 252, respectively (Table 1).

**Table 1.** Characteristics of provinces out of Hubei (median, inter-quartile range)

	All provinces (*N* = 22)	Eastern provinces (*N* = 11)	Central provinces (*N* = 6)	Western provinces (*N* = 5)
COVID-19 case number	459 (245−935)	346 (168−760)	962 (482−1018)	252 (245−539)
Migration scale index from Hubei	1.37 (0.88−3.45)	1.20 (0.38,2.41)	3.86 (0.68−10.09)	2.35 (1.03−2.45)
BI	1992.38 (1453.74−3348.19)	2535.6 9(1757.19−4129.42)	2083.22 (1463.47−2488.15)	1441.95 (1367.21−1525.24)
Population ≤14 years old (thousand)	814.94 (508.73−1269.42)	729.09 (23 479−136 008)	1105.93 (580.01−1269.42)	766.80 (602.78−1098.50)
Population 15–64 years old (thousand)	3163.50 (2476.80−5304.31)	3138.48 (1649.96−5845.03)	3741.26 (2939.17−4787.91)	2832.31 (2476.80−3349.68)
Populaton ≥ 65 years old (thousand)	456.02 (356.4−873.64)	374.40 (269.25−891.61)	639.33 (455.50−841.68)	443.59 (428.90−477.82)
Male/female ratio (%)	103.95 (101.90−108.30)	103.50 (102.70−11 110	104.70 (103.80−108.30)	101.90 (99.60−107.20)
Registered doctors per 1000 population	157.05 (135.34−303.10)	180.92 (154.62−303.10)	153.02 (116.65−426.20)	135.37 (135.31−156.85)
Registered nurses per 1000 population	211.85 (196.81−233.15)	232.75 (196.82−274.15)	198.67 (184.73−208.97)	224.97 (216.16−225.74)
Beds in infectious disease department per 1000 population	8.44 (7.26−10.11)	7.26 (6.53−9.80)	9.23 (8.54−11.14)	8.35 (7.38−10.11)
Public health professionals per 1000 population	9.12 (7.61−11.31)	8.39 (7.32−9.57)	9.59 (8.55−10.09)	11.87 (11.31−12.02)
Per capita GDP (Yuan)	55 478.50 (47 712.00−91 197.00)	91 197.00 (58 008.00−120 711.00)	47 573.00 (45 328.00−50 152.00)	48 883.00 (41 489.00−63 477)
Per capita health care expenditure (Yuan)	1410.15 (1135.90−1704.80)	1510.90 (1319.50−2390.00)	1289.75 (1135.90−1424.00)	1320.20 (1075.60−1471.90)

**Fig. 2.**
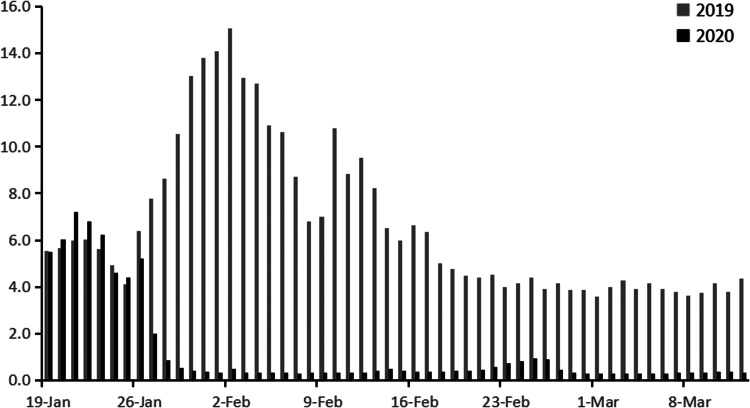
Migration scale index out of Hubei from 19 January 2019 to 12 March 2019 and from 19 January 2020 to 12 March 2020.

**Fig. 3.**
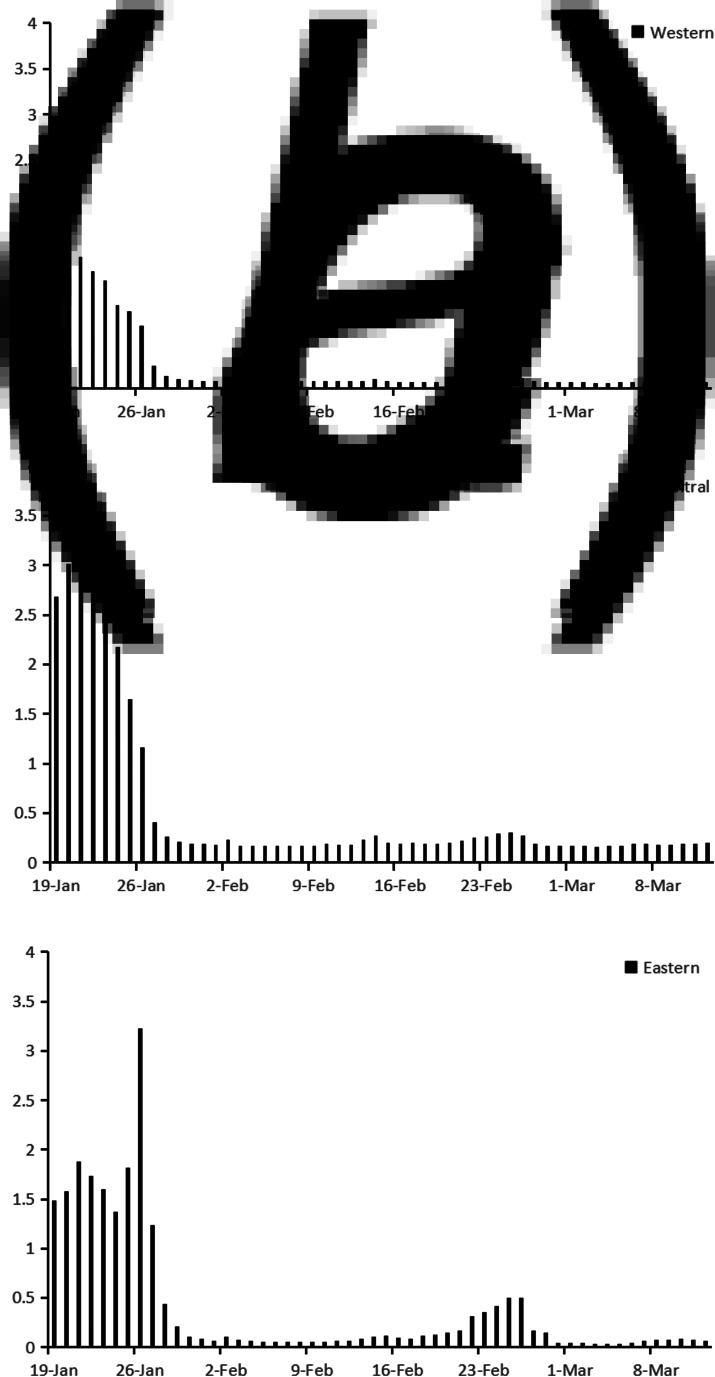
Migration scale index from Hubei to eastern, central or western province from 19 January 2020 to 12 March 2020.

Migration scale index out of Hubei from 19 January to 12 March are shown in Figure 2. Before 23 January 2020, migration scale index out of Hubei was higher than 6, which was similar to that in 2019. On 24 January, the Chinese government locked down 14 cities in Hubei due to the quick spread of COVID-19, when migration scale index dropped to less than 5. In order to contain SARS-CoV-2 spreading further, the whole province was locked down before 27 January. Then, the index plunged to less than 1, but the data were experiencing an upward trend in 2019. Until 12 March, the index stayed at around 0.4, which was much lower than that in 2019. Figure 3 showed the change of migration scale index from Hubei to eastern, central and western provinces. The peak value of migration scale index was much lower in western provinces than that in eastern or central provinces, as well as the total migration scale (Table 1).

**Fig. 4.**
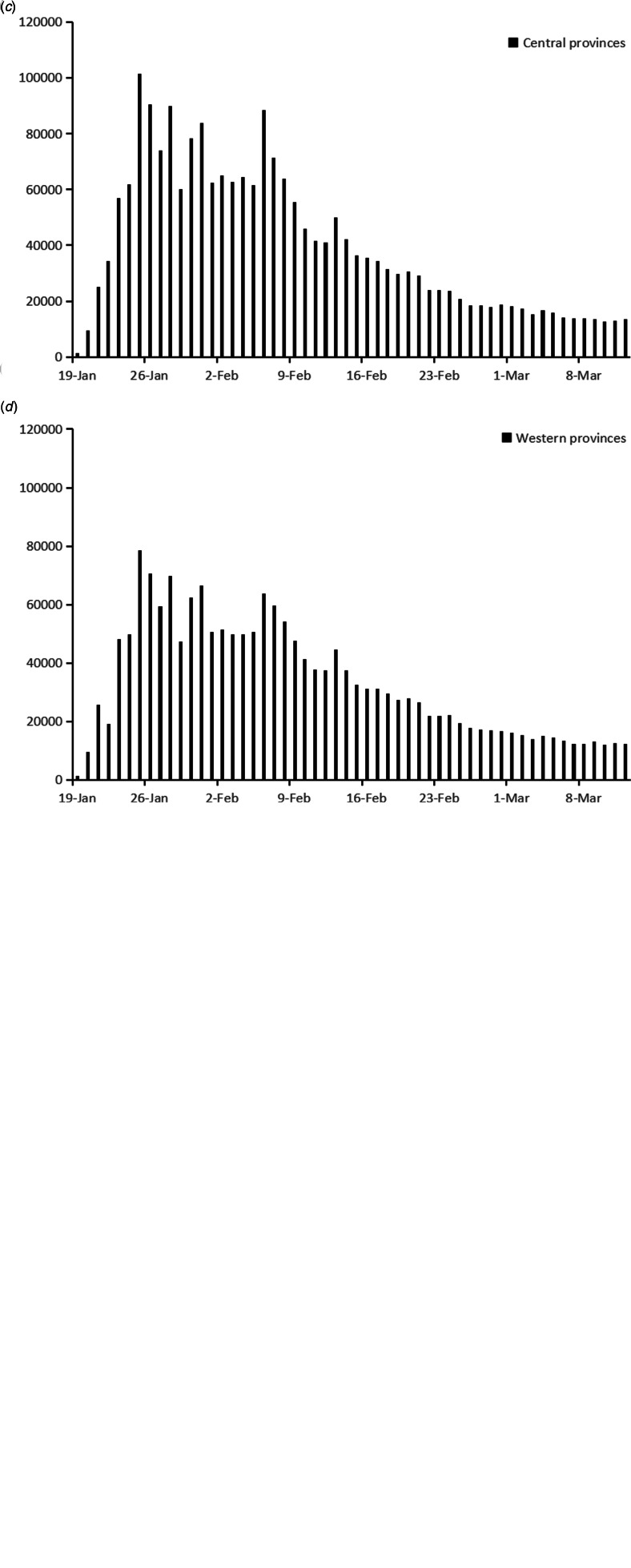
BI for COVID-19 from 19 January 2020 to 12 March 2020.

BI from 19 January 2020 to 12 March is shown in Figure 4. Before 19 January, BI for COVID-19 was less than 10 000 in China. After that, it surged from 34 506 on 19 January to the top of 2 490 679 on 25 January. Then the index decreased gradually to around 500 000 at the end of February and fluctuated between 400 000 and 500 000 before 12 March. Among the eastern, central and western provinces, change patterns of BI were similar, but in western provinces the value of BI was lower (Fig. 4 and Table 1).

The annually reported data of eastern, central and western provinces were shown in Table 1. Medians of population by age groups were highest in central provinces, when sex ratios were similar among the three regions. With regard to public health resource per 1000 population, the medians of registered doctors and nurses were highest in eastern provinces, while the western provinces owned the highest median of public health professionals. With regard to economic status, eastern provinces had significantly higher per capita GDP and health care expenditure.

### Correlation analysis

Pearson's correlation analysis was applied to migration scale index and BI, which showed significant correlation (*r_x_*_,*y*_ = 0.4262, *P* < 0.0001). Spearman's rank-order correlation was conducted among annually reported variables. The results are shown in Table 2. Population ≤14 years old was significantly correlated with population of other two age groups and per capita health care expenditure. Population of 15–64 years old was significantly correlated with population ≥65 years old. As for variables related to public health resource, the number of registered nurses was significantly correlated with public health professional number. And the correlation between per capita GDP and per capita health care expenditure was significant, too. Therefore, daily and annually reported variables, except cumulative BI, population ≤14 years old, population of 15–64 years old, registered nurse and per capita health care expenditure, were included into multivariate regression. In addition, interaction items among public health professionals, registered doctors and beds in infectious department were included, because their roles and effects were related in preventing and controlling COVID-19 pandemic. Public health professionals and doctors both participated in epidemic investigation and COVID-19 testing, while doctors and beds in infectious disease department reflected the capacity of COVID-19 admissions.

**Table 2.** Spearman's rank-order correlation analysis among annually reported variables (*r_x_*_,*y*_, sig.)

	Population 15−64 years old (thousand)	Population ≥ 65 years old (thousand)	Male/female ratio (%)	Registered doctors (for per thousand people)	Registered nurses (for per thousand people)	Beds in infectious disease department (for per thousand people)	Public health professionals (for per thousand people)	Per capita GDP (Yuan)	Health care per capita expenditure (Yuan)
Population ≤14 years old (thousand)	0.92707 <0.0001	0.83749 <0.0001	0.13920 0.5367	0.13721 0.5426	−0.39890 0.0659	−0.07166 0.7513	−0.37225 0.0880	−0.33810 0.1238	−0.47658 0.0249
Population 15–64 years old (thousand)		0.90493 <0.0001	0.15053 0.5037	0.09405 0.6772	−0.37894 0.0820	−0.05885 0.7948	−0.39004 0.0727	−0.15470 0.4918	−0.32455 0.1406
Population ≥ 65 years old (thousand)			−0.12499 0.5794	0.11991 0.5950	−0.31983 0.1468	−0.03186 0.8881	−0.34391 0.1171	−0.13369 0.5531	−0.28436 0.1996
Male/female ratio (%)				−0.19218 0.3916	−0.34767 0.1129	0.12035 0.5937	−0.33774 0.1242	0.05386 0.8118	−0.02515 0.9115
Registered doctors (for per thousand people)					0.11405 0.6133	−0.15095 0.5025	0.15081 0.5029	−0.12448 0.5810	−0.19218 0.3916
Registered nurses (for per thousand people)						−0.23968 0.2827	0.97539 <0.0001	0.08504 0.7067	−0.04659 0.8369
Beds in infectious disease department (for per thousand people)							−0.20387 0.3628	−0.264553 0.2342	0.00654 0.9770
Public health professionals (for per thousand people)								−0.06181 0.7847	−0.13869 0.5382
Per capita GDP (Yuan)									0.74027 <0.0001

### Regression splines

Spline regression model was applied to fit cumulative COVID-19 case number in eastern, central, western and all provinces, respectively. The results are shown in Table 3. Models with knots and covariates fitted the data better than the ones with knots only. No quadratic or cubic terms were included. In the model of all provinces, estimated knots were 8th and 24th days, which were similar with knots of eastern and central province models. But in the model of western provinces, knots were located at 7th and 31st days. As for covariates, migration scale index from Hubei was included in each model for eastern, central and western provinces, which was positively correlated with cumulative COVID-19 case number. Other included covariates were different among the three regions.

**Table 3.** Spline regression of cumulative COVID-19 case number in eastern, central, western and all provinces

Model	Variables	Estimates	s.e.	Adjusted *R*^2^	AIC
Model 1^a^	D5	27.9	1.02	0.6596	13 721.5264
	D23	−25.26	2.02		
Model 2^b^	D8	21.98	1.14	0.8702	12 581.3221
	D24	−21.41	1.69		
	Migration scale index from Hubei	93.7	3.04		
	Registered doctors	−0.09	0.009		
	Registered nurses	−3.05	0.62		
	Beds in infectious disease department	−8.11	1.45		
	Populaton ≥ 65 years old	0.12	0.02		
Model 3^c^	D4	21.65	1.09	0.6112	6897.3392
	D28	−21.53	2.87		
Model 4^d^	D8	18.89	1.62	0.8537	6320.9008
	D24	−17.87	2.42		
	Migration scale index from Hubei	116.29	4.61		
	Public health professionals	−3.07	0.63		
	Populaton ≥ 65 years old	0.09	0.02		
	Per capita GDP	−0.0004	0.0002		
Model 5^e^	D5	38.45	1.71	0.8099	3725.9779
	D25	−36.69	3.71		
Model 6^f^	D8	37.82	1.75	0.9461	3320.5095
	D25	−39.77	2.67		
	Migration scale index from Hubei	91.84	3.44		
	Registered doctors	−0.08	0.009		
	Public health professionals	−19.09	2.13		
Model 7^g^	D4	14.29	0.65	0.8025	2665.7809
	D29	−14.67	1.80		
Model 8^h^	D7	8.54	0.63	0.9400	2352.1051
	D31	−9.31	1.28		
	Migration scale index from Hubei	62.53	3.25		
	Registered doctors	−0.28	0.04		
	Population ≥ 65 years old	0.1	0.01		

a Model with knots fitting cumulative COVID-19 case number in all provinces.

b Model with knots and covariates fitting cumulative COVID-19 case number in all provinces.

c Model with knots fitting cumulative COVID-19 case number in eastern provinces.

d Model with knots and covariates fitting cumulative COVID-19 case number in eastern provinces.

e Model with knots fitting cumulative COVID-19 case number in central provinces.

f Model with knots and covariates fitting cumulative COVID-19 case number in central provinces.

g Model with knots fitting cumulative COVID-19 case number in western provinces.

h Model with knots and covariates fitting cumulative COVID-19 case number in western provinces.

## Discussion

**Fig. 5.**
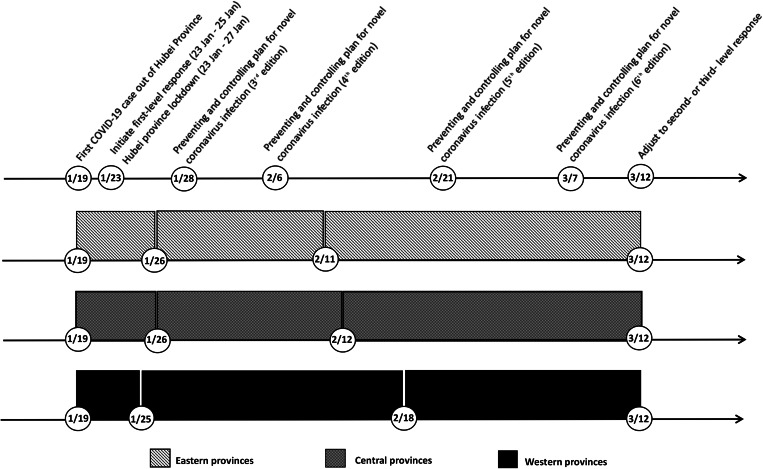
Timeline of implemented measures in China and the three stages in eastern, central and western provinces.

In this study, COVID-19 case number out of Hubei during first-level response was analysed with spline regression model. A stepwise procedure was used to select the number, location and degree of polynomial, as well as covariates. In order to explore the change patterns of COVID-19 case number in different regions, spline models were used to fit the data of eastern, central and western provinces, respectively. The research period was divided into three stages by two knots in all models, but the locations of knots were different in the three regions (Fig. 5).

The first stage was similar among the three regions, lasting from 19 January to around 26 January. In this stage, cumulative COVID-19 case number was associated with covariates, such as migration scale index out of Hubei, public health professionals and registered doctors, but not the number of days since 19 January. The daily situation reports of Guizhou, Tianjin and Shanghai, which released the exposure history of each COVID-19 case, provided an insight into this situation [25–27]. In the first 8 days of outbreak, imported cases mainly contributed to the increase of COVID-19 case number, taking a proportion of 50.0%~88.9%. In order to prevent case spilling over, Hubei province was gradually locked down since 23 January, which decreased the risk of case importation for other provinces. In the second stage, cumulative COVID-19 case number started to increase rapidly with time. Imported case proportion decreased, and indigenous case proportion surged, ranging from 43.7% to 73.5%. In this stage, SARS-CoV-2 spread among inhabitants and secondary infection cases were diagnosed extensively. The Chinese government took a combination of preventing and controlling measures to contain the pandemic. In eastern and central provinces, the growth slowed down since 11 or 12 February. However, in western provinces, it slowed down after 18 February. Cumulative COVID-19 case number of western provinces increased more slowly in the second stage, while eastern and central provinces experienced a sharper increase in a shorter second stage.

Previous studies mostly fitted the data of COVID-19 with regression models at national or global level. Ekum *et al*. [28] conducted hierarchical polynomial regression with daily cases of COVID-19 globally, which indicated cubic model fitted the data better than linear or quadratic models. Demertzis *et al*. [16] applied spline model and cubic model to the cumulative number of COVID-19 infections, deaths and ICU patients in Greece. In their study, spline models outperformed cubic ones with each outcome. Sousa *et al*. [15] and Agiwal *et al*. [14] fitted the daily reported COVID-19 cases of European, American and Asian countries with linear and cubic spline terms, identifying the knots of each country. However, change patterns might be different among various regions in a country. In this study, models with different knots and parameters were developed for eastern, central and western provinces, which showed the specific change patterns of COVID-19 among the three regions. Therefore, in addition to studies at global and national levels, analysis with province data was needed. The difference might be attributed to regional risk of case importation, local epidemiological features, applicability of preventing and controlling measures and public compliance. To reveal the cause of different change pattern at province level, studies with more detailed data on case characteristics, implementation of controlling measures and public behaviours were required.

Meanwhile, previous studies exploring the knots of COVID-19 case number did not include other explanatory variables. In this study, stepwise procedure was used to select knot number, locations and covariates. There were different systematic methods to estimate the number and locations of knots. Friedman [29] and Silverman *et al*. [30] discussed stepwise-based method, Osborne *et al*. [31] proposed an algorithm based on the LASSO estimator and Denison *et al*. [32] considered the bayesian method. All these methods were successful. Stepwise procedure allowed for any combination of adjustments (linear, quadratic and cubic) in each segment and took into account other explanatory variables at the same time. It provided the added utility of a simple, unconstrained function which can be easily implemented in non-linear trend analysis. This study showed the models with knots and covariates fitted the data better than the models with knots only. In addition to the segments of time, variance of cumulative COVID-19 case number was attributed to transient population from Hubei, population ≥65 years old and variables reflecting health resources at the province level. These findings were consistent with other studies' result. Chen's study showed migration scale index from Hubei was associated with confirmed COVID-19 cases per day in China except Hubei from 20 January to 2 February [33]. Data from seven countries, including Spain, Canada, Netherlands, Italy, Germany and Korean, revealed that older population with low immunity had higher risk of infection, taking 27%–58.1% of the confirmed cases [34–39]. A comparison analysis between COVID-19 and H1N1 outbreaks proved public health professionals and doctors played a crucial role in public health emergency response [40, 41]. These findings supported the assumption; spline regression models with other explanatory variables might provide better insight into the trend pattern of COVID-19 cases.

This study had several potential limitations. Firstly, the cumulative number of COVID-19 cases did not equal to the total number of infections due to unconfirmed cases. Asymptomatic cases might not be recognised due to the lack of symptoms and infected patients could ignore the mild symptoms at early stage resulting in delay to diagnosis. Secondly, the data extracted from the Chinese Health Statistical Year book was based on the latest available national survey finished in 2018, which might not match the medical resources and population characters during outbreak period completely. Thirdly, some covariates related to the spread of SARS-CoV-2, such as testing intensity, were not included in this study, because no corresponding public database were available.

## Conclusion

This study analysed cumulative COVID-19 case number during the first-level response with spline regression models at province level out of Hubei. Inclusion of covariates made spline models fit COVID-19 data better. The research period was divided into three stages by two knots. The spline models of eastern, central and western provinces demonstrated different trend patterns in each region. Spline function with covariates would be a practical tool to analyse the non-linear trend of COVID-19 data. Analysis at province level was necessary to explore the change patterns of COVID-19 data in corresponding regions. To reveal more information about the regional characteristics of COVID-19 pandemic, studies with more detailed data at province level were needed. A better understanding of COVID-19 pandemic will help the policy makers to more effectively control the spread of SARS-CoV-2 and lessen the adverse social effect to a greater extent.

## Data Availability

The datasets used in this study is available from the corresponding author Chen Liang on reasonable request (Email: liangchen_sjtu@163.com).
